# Influence of Organic Matter and Speciation on the Dynamics of Trace Metal Adsorption on Microplastics in Marine Conditions

**DOI:** 10.3390/toxics12110820

**Published:** 2024-11-16

**Authors:** Ana Rapljenović, Marko Viskić, Stanislav Frančišković-Bilinski, Vlado Cuculić

**Affiliations:** 1 Laboratory for Physical Chemistry of Traces, Division for Marine and Environmental Research, Ruđer Bošković Institute, Bijenička c. 54, 10000 Zagreb, Croatia; arapljen@irb.hr (A.R.); mviskic@irb.hr (M.V.); francis@irb.hr (S.F.-B.); 2 Selvita Ltd., Prilaz Baruna Filipovića 29, 10000 Zagreb, Croatia

**Keywords:** microplastics, humic acid, trace metals, seawater, speciation modeling, adsorption

## Abstract

Dissolved organic matter (DOM), primarily in the form of humic acid (HA), plays a crucial role in trace metal (TM) speciation and their subsequent adsorption dynamics on microplastics (MP) in aquatic environments. This study evaluates the impact of environmentally relevant concentrations of HA on the adsorption behaviors of essential (Co, Cu, Ni, and Zn) and toxic (Cd and Pb) TMs onto polyethylene (PE) and polypropylene (PP) pellets, as well as PP fibers under marine conditions, during a six-week experiment. The HA concentrations were 0.1, 1, and 5 mg/L, while all metals were in the same amounts (10 µg/L). Results reveal that HA significantly influences the adsorption of Cu, Pb, and Zn on MP, particularly on PP fibers, which exhibited the greatest TM adsorption dynamics. The adsorption patterns correspond to the concentrations of these metals in seawater, with the sequence for pellets being Zn > Cu > Pb > Ni > Co~Cd, and for fibers Cu > Zn > Pb > Co~Ni > Cd. Speciation modeling supported these findings, indicating that Cu, Pb, and Zn predominantly associate with HA in seawater, facilitating their adsorption on MP, whereas Cd, Co, and Ni mainly form free ions and inorganic complexes, resulting in slower adsorption dynamics. Statistical analysis confirmed the influence of HA on the adsorption of Cd, Pb, Cu, and Ni. By investigating the dynamics of TM adsorption on plastics, the influence of DOM on these two contaminants under marine conditions was evaluated. The presented results can help in forming a better understanding of synergistic plastic and trace metal pollution in marine systems that are relevant at the global level, since both contaminants pose a serious threat to aquatic ecosystems.

## 1. Introduction

Microplastics (MPs) are at the focus of recent intensive research as stable and persistent pollutants of estuarine and marine environments [1,2]. Plastic debris represents approximately 80% of all pollutants of the aquatic environment, with ever increasing amounts. The polymers considered in this work are the most abundant components of microplastics found in the Mediterranean Sea, with PE covering the majority of microplastic debris (52–67%), and PP as the second most common (16–17%) polymer [3,4,5].

Microplastic particles formed by mechanical, chemical, and other forms of degradation of plastic litter are capable of accumulating substantial amounts of trace metals (TM) from the environment [6,7,8,9,10,11,12,13,14,15,16,17,18,19,20,21]. TMs such as Zn, Cu, Ni, and Co are essential micronutrients at low concentrations, but can become toxic in higher amounts [22,23]. Non- essential metals like Pb are highly toxic, even at low concentrations, as well as Cd, which can be hazardous despite its micronutrient-like distribution in marine waters [24,25]. In Directive 2008/105/EC [26], Cd, Pb, and Ni were listed as priority substances, with the aim of progressively reducing pollution from those metals. Plastics in marine environments act as new substrates for TM adsorption, influencing their distribution and bioavailability [7]. TMs, adsorbed to the surfaces of MPs, enter organisms via ingestion, gradually accumulating within the body and significantly impacting the survival of marine organisms. These TM have been shown to cause toxic physiological effects and even mortality in various species [27,28]. Anthropogenic activities contribute to the elevated presence of both TM and plastics in marine ecosystems, leading to unknown and potentially far-reaching consequences [20]. Accumulation of TMs on MPs proceeds according to two pathways, either by direct bonding with functional groups exposed on the polymer surface [29], or by prior interaction with free dissolved organic matter (DOM) or biofilms formed on surfaces exposed to aquatic ecosystems [7,30,31]. The adsorption process may occur rapidly or slowly, with TM adsorption equilibrium in some cases not being achieved within a year [9]. Apart from the active sites on polymer surfaces, TM adsorption can be enhanced by structural and chemical changes caused by the weathering of plastics, which increases the formation of oxidized functional groups and the specific area available for metal binding [10].

Humic substances (HSs) are major components of natural organic matter (NOM) in surface waters. They make about 50% of DOM and exert an important effect on the speciation and adsorption behavior of TMs in the environment [32,33]. Humic substances contain carboxylate and phenolate groups [34], mostly responsible for metal chelation or complexation [35]. Metal uptake is further facilitated by the basic pH of seawater due to the greater ionization of HS functional groups. In addition, such conditions favor repulsion of charged groups in HS and an increase in their molecular size, leading to enhanced binding [36]. DOM can also interact with synthetic polymers by binding to microplastics’ surfaces, mostly through chemisorption [37,38]. MP with formed HA layers has potential to accumulate larger quantities of TM when compared to intact MPs due to the increase in their structural inhomogeneity or surface negative charge density [39]. Altering the bioavailability of metals, HS can significantly impact the biome [40,41,42], and potentially increase toxic effects on aquatic biota through the formation of MP–HS–metal complexes that enter the food chain, facilitated by MPs’ large specific surface areas [43]. Accumulation of TMs on microplastics in the presence of DOM is influenced by simultaneous competition of different metals for binding sites and by an additional equilibrium defined by the complex formation before or after contact with MPs. Seawater conditions, such as pH and ionic strength, define the metal speciation, and, together with charge and the electric bilayer on the MP’s surface, influence adsorption outcome.

Most of the papers published so far used a simplified approach, with metal–aqueous solutions or artificial seawater, which only partially meets the conditions for TM adsorption in natural waters. Up to the present day, very few papers assessed the influence of natural organic ligands from seawater on the process of adsorption of metals onto MPs. A recent study reported on the adsorption of Pb on virgin and biofilm-covered polyvinyl chloride (PVC) in the presence of HA [44], while others showed that the adsorption of Cu on polystyrene (PS) in the presence of DOM was fastest within the first 48 h [45]. Adsorption of Cu, Ni, and Zn onto nylon in the presence of fulvic acid (FA) was reported to be weak on virgin MPs [31], while in the case of weathered nylon, added FA enhanced the adsorption of Ni due to formation of labile organic complexes. Moreover, strongly decreased adsorption was found for Cu when the highest amounts of FA were added, probably due to formation of stabilized Cu-FA complexes in the solution. Furthermore, it is shown that the amounts of adsorbed Pb on PS increased with HA, with a fast rate of metal binding to HA and subsequent adsorption [46]. In addition, their spectral studies showed a significant increase in inhomogeneity of the MP surface due to HA binding and distribution. A previous study reported that the increase in added HA led to an increase in adsorbed Cd, presumably due to the influence of the polymers specific surface area and surface charge [47]. Also, Cd adsorption took place in three stages, during the interaction between metal ions and MP, saturation, and diffusion, followed by the equilibrium being reached within 24 h.

This study was designed to develop and conduct a prolonged model experiment investigating the influence of humic acid on the adsorption of trace metals onto PP fibers and PP and PE pellets, commonly found pollutants in seawater. The effects of three humic acid concentrations that mimic those naturally occurring in natural aquatic systems [48] were specifically evaluated. Furthermore, the interactions between trace metals and humic acid and their adsorption onto microplastics was supported by statistical analysis and speciation modeling to understand the dynamic relationships in these processes.

## 2. Materials and Methods

### 2.1. Chemicals and Samples Preparation

For this study, 1 g/L stock solutions of Cu(NO_3_)_2_, Ni(NO_3_)_2_, Co(NO_3_)_2_, Zn(NO_3_)_2_, Cd(NO_3_)_2_, and Pb(NO_3_)_2_, with perchloric and nitric acids, sodium acetate, 1,2-cyclohexanedione dioxime (nioxime), sodium hydroxide (30%, *w*/*w*), humic acid, and sodium tetraborate decahydrate salt were used (Merck, Darmstadt, Germany). All mentioned reagents except HA were of Suprapur^®^ grade. Type I reagent grade Milli-Q^®^ water (Merck Millipore) was used for all reagent preparations and rinsing. All bottles, cuvettes, flasks, strainers, tweezers, and other pieces of laboratory equipment used for experiment setup and sampling were cleaned and rinsed with nitric acid (10%, *w*/*w*) and Milli-Q^®^ water prior to use.

Bottles used for the adsorption model experiment (2 L), as well as bottles for individual sampling (250 mL) were made of fluorinated ethylene propylene (FEP), and perfluoroalkoxy alkane (PFA) (NALGENE^®^, Thermo Fisher Scientific, Waltham, MA, USA) to prevent loss of material due to potential TM adsorption on the bottles’ walls and to enable direct UV irradiation of the seawater in these vessels.

The high-density polyethylene (HDPE) pellets used for model experiments were purchased from Borealis AG (Vienna, Austria), and polypropylene (PP) pellets were purchased from TVK (Tiszaújváros, Hungary), while PP fibers were purchased from Belgian Fibers Group NV (Mouscron, Belgium). The physical features of the plastic microparticles were, for PE pellets, as follows: unit mass 0.018 g, density 0.96 g/cm^3^; for PP pellets: unit mass 0.039 g, density 0.90 g/cm^3^; while for both pellets, average length and width ranged between 4.0 and 4.3 mm and 2.1 and 2.7 mm, respectively. For PP fibers, unit length was 18 mm, diameter was 34 μm, and density was 0.91 g/cm^3^.

HA stock solution (1 g/L) was prepared by adding 50 mg of HA standard and 50 µL of Suprapur^®^ grade NaOH (30%, *w*/*w*) to 50 mL of Milli-Q^®^ water in a flask and sonicating (40 kHz, 240 W peak ultrasonic output) the mixture in an ultrasonic bath (emmi EMAG AG, Mörfelden-Walldorf, Germany) for 60 min at room temperature. HA stock solution was tested for the presence of any TM. To 100 mL of Milli-Q^®^ water, 100 µL of HA stock solution was added together with 200 µL of concentrated HNO_3_. This solution was UV irradiated for 24 h and analyzed for TM content. Recorded TM concentrations were negligible (≤1%) in comparison to the mass of metals adsorbed on microparticles, and hence did not significantly alter the measured quantities of adsorbed TMs.

A standard solution of Cd(II), Pb(II), Cu(II), Zn(II), Ni(II), and Co(II) (concentration of individual metals, 10 mg/L) was prepared by diluting the commercial single metal stock solutions in 10 mL of Milli-Q^®^ water to the final TM concentrations (10 µg/L) of the experiment. A fresh standard solution of TM was prepared prior to each model experiment. Prior to model experiments, the influence of HA addition on the seawater pH was tested. The pH was monitored continuously in 10 mL of seawater during the gradual addition of 100 µL of HA stock solution in 10 µL increments.

For the assessment of TM adsorption on MP particles under the influence of natural organic ligands, seawater was sampled at two locations on the Adriatic Sea coast at areas without significant anthropogenic influence: Ploče area (southern Adriatic Sea) and Medveja Bay (northern Adriatic Sea), and stored in a refrigerator at 4 °C. Just before the adsorption experiment, seawater was filtered (cellulose nitrate filter, pore size 0.45 µm, Sartorius, Göttingen, Germany), and UV irradiated under a 150 W medium pressure (entire UV spectrum, emission peak in the UVA range at 366 nm) mercury lamp (Hanau, Germany) for 48 h to degrade all present organic matter and microorganisms that could compete with added HA for TM. The salinity of seawater was measured by S-10E refractometer (Atago, Tokyo, Japan) and the pH was determined by an ATI Orion 320 pH meter (Thermo Fisher Scientific, USA). The pH value and salinity of seawater used for the model experiment was determined after seawater filtration and UV irradiation, prior to the experimental run. Additionally, the pH of the seawater was assessed after the first and the final sampling. Parameters of seawater samples can be found in Table 1.

Different amounts of HA (0.1, 1, and 5 mg/L) were added to each of the three seawater solutions, while HA was not present in the blank. All seawater solutions (including blank) contained 10 µg/L of each TM.

### 2.2. Adsorption Experiment

**Figure 1:**
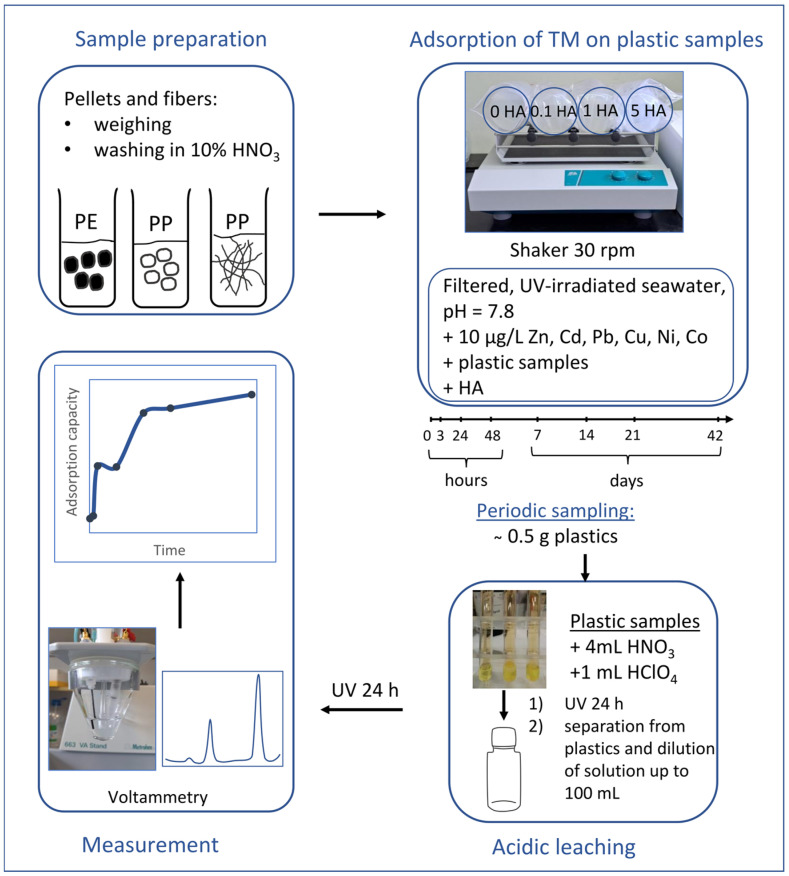
Flowchart of adsorption experiment, leaching and measurement of TM.

The adsorption experiment was set up in 2 L FEP bottles by placing 1 L of filtered and UV irradiated seawater together with 10 g of pre-weighted plastic MPs in each bottle. Prior to addition, plastic particles were washed with 10% nitric acid and rinsed with Milli-Q^®^ water. To each seawater–microplastic mixture, final HA concentrations (0.1 mg/L, 1 mg/L, and 5 mg/L) were added, except for the bottle with the control (blank) solution. Subsequently, 10 µg/L of each TM was added, including the blank bottle. The FEP bottles were placed on a stirrer and constantly shaken at 30 rpm. The flow chart of the experiment is presented in Figure 1.

The experiments were run for a total of 42 days. The microplastics in each bottle were sampled at given time intervals, with seven samplings after 3 h, 1 d, 2 d, 7 d, 14 d, 21 d, and 42 d. An additional sampling was performed for the fiber experiment at hour 0, immediately after the addition of all the components. The experimental time from 0 h to 21 d is referred to as the first stage of the experiment, while the period after the 21st day until the end of the experiment is referred to as the second stage. The sampling is performed by separating approximately 0.5 g of particles, which were placed in quartz glass cuvettes. In addition, with each sampling of the plastics, 50 mL of solution was extracted to maintain a consistent plastic-to-solution ratio. An acid-leaching procedure was performed by adding 4 mL of nitric and 1 mL of perchloric acid (both Suprapur^®^ grade), to the microplastic samples. The cuvettes were shaken, and UV irradiated overnight. The leachate was transferred quantitatively to the glass conical flask of 100 mL using a micropipette, diluted with Milli-Q^®^ water, and transferred to FEP bottles, which were placed under UV light for 24 h. The fibers were rinsed with Milli-Q^®^, dried for 48 h at 60 °C, and weighed afterwards. Acid control (blank) solutions were also prepared by addition of concentrated acids to empty cuvettes, and following treatment as described above. During the experiment, the room temperature was monitored daily, and ranged slightly from 19.0 °C to 21.0 °C. The control (blank) samples containing seawater, plastic MPs and the TM standard solution without HA were simultaneously measured.

### 2.3. Trace Metal Analysis

The mass fractions of TM (Cd, Cu, Pb, Zn, Ni, and Co) adsorbed on plastic MPs and their concentration in model solutions and seawater samples were determined using differential pulse stripping voltammetry by µAUTOLAB potentiostat/galvanostat PGSTAT204 (Metrohm Autolab, Utrecht, The Netherlands). The measuring system was connected with a Metrohm 663 VA Stand three-electrode system, while the working electrode used was a static mercury drop electrode (SMDE). To an electroanalytical cell, 15 mL of the sample was added, and the pH value was adjusted to 2 with NaOH addition. Differential pulse anodic stripping voltammetry (DPASV) with a standard addition method was used for Cu, Cd, Pb, and Zn analyses [49,50] for measurement in water and solid samples. For the measurement of Zn, the pH was adjusted to 4, with addition of sodium acetate solution. Voltammetric parameters were as follows: modulation time 0.04 s, interval time 0.1 s, modulation amplitude 20 mV, and step potential 2 mV. The deposition times (*t*_d_) were 300 or 600 s at deposition potential (*E*_d_) = −0.85 V for Cu, Cd, and Pb, while for Zn *t*_d_ was 60 s at *E*_d_ = −1.2 V. Adsorptive cathodic stripping voltammetry (ACSV) with a standard addition method was used to measure concentrations of Ni and Co. For the measurement of Ni and Co, 12 mL of the sample was placed in a cell, and the pH was adjusted to 8 by addition of NaOH solution and borate buffer, and nioxime ligand (15 µL, 0.01 mol/L) solution was added. Voltammetric parameters were as follows: modulation time 0.04 s, interval time 0.1 s, modulation amplitude 20 mV, step potential 2 mV, and *t*_d_ 120 s at *E*_d_ = −0.75 V. The adsorbed quantities of TM are expressed as a mass fraction of metal per weight of sampled microplastics. Electrochemical characteristics (redox potential, peak current, half-peak width) of the obtained voltammetric signals (peaks) were analyzed using the ECDSOFT (ElectroChemical Data SOFTware 2.0) software [51].

### 2.4. Speciation Modeling

TM speciation in seawater (Table 2) was calculated using Visual MINTEQ 3.1 software according to the major and trace element composition [52]. TM interactions with organic matter were estimated with the NICA-Donnan humic model (NDHM) implemented in the software. Humic acid (HA) parameters given by the software were used together with the added dissolved HA concentrations 0.1 mg/L, 1 mg/L, and 5 mg/L in previously filtered and UV-irradiated seawater. Parameters for DOC were set to the ratio of active DOM to DOC by a factor of 2, according to [53].

### 2.5. Quality Assurance and Quality Control (QA/QC)

The limits of quantification (LOQ) in aquatic solutions were 1 ng/L, 5 ng/L, 2 ng/L, and 10 ng/L for Cd, Cu, Pb, and Zn, respectively (anodic stripping voltammetry), while LOQ for Ni and Co (cathodic stripping voltammetry) were 10 ng/L and 1 ng/L, respectively (10 σ rule with 10 min deposition time; standard addition method, pH < 2), assessed in Milli-Q^®^ water. The precision of the applied method (quality control) for Cd, Pb, Cu, Zn, Co, and Ni was checked measuring the standard reference material (SRM) NASS-6 from National Research Council of Canada for TM in ocean seawater, with certified values (µg/L): Cd 0.0311 ± 0.0019, Pb 0.006 ± 0.002, Cu 0.248 ± 0.025, Zn 0.257 ± 0.02, Co 0.015, and Ni 0.301 ± 0.025. The accuracy of TM analysis in solid samples was tested measuring the SRM 2702 of National Institute of Standards & Technology, with certified values (mg/kg): Cd 0.817 ± 0.011, Pb 132.8 ± 1.1, Cu 117.7 ± 5.6, Zn 485.3 ± 4.2, Co 27.76 ± 0.58, and Ni 75.4 ± 1.5. All determined concentrations and mass fractions were measured in triplicate and were found to be within 5% of certified values. All reported results (TM concentrations and mass fractions) were statistically tested and presented with 95% confidence intervals (c.i.), calculated by StandAdd software 2.0 (DarOma_Soft software package).

### 2.6. Statistical Analyses

The program Statistica 6.0 [54] was used for all statistical analyses. Basic statistical parameters were determined (N—number of cases, mean, median, sum, minimum, maximum, range, and standard deviation) to provide a deeper understanding of the experimentally determined values, without presenting the entire dataset. To determine the strength of the linear correlation of mass fractions between researched elements, correlation analysis was performed by calculating Pearson’s correlation coefficient and presenting it as a correlation matrix. The values obtained were statistically significant at *p* < 0.05. The boxplot method was used to determine anomalies in the samples. The empirical cumulative distribution plots are utilized to construct normal or lognormal boxplots. The box length was of interquartile range, where outlier values were defined between 1.5 and 3 box lengths from the upper or lower edge of the box. Extreme values exceed three box lengths from the edge of the box [55,56].

To assess how HA concentration affects the adsorption of each metal, regression analysis was used. The regression analysis and all its aspects are described in detail by [57].

## 3. Results and Discussion

The adsorption behaviors of trace metals on PE and PP pellets, as well as PP fibers were studied at different humic acid concentrations. Basic statistical parameters (N, Mean, Median, Sum, Minimum, Maximum, Range, and Std. Deviation) were calculated for each of three datasets (PE pellet, PP pellet and Fibers) separately and presented in Table S1 (in Supplementary Materials). For all observed elements there is a large difference between the pellet samples (both PE and PP) and those of fiber samples—fibers have a much higher value of concentration of all elements, and for some elements for the order of magnitude. There are differences between the two types of pellets, with PP pellets having a significantly higher concentration of elements than PE pellets. The only exception is Ni, where there is a lower concentration in PP pellets, but the difference is not very high. To obtain insight into statistical anomalies and distribution, scatterboxes with boxplots were constructed for each of three datasets (PE pellets, PP pellets, and fibers) separately. Results are presented in the Supplementary Materials (Chapter S1, Figures S1–S3).

### 3.1. Adsorption of TM on PE Pellets

**Figure 2:**
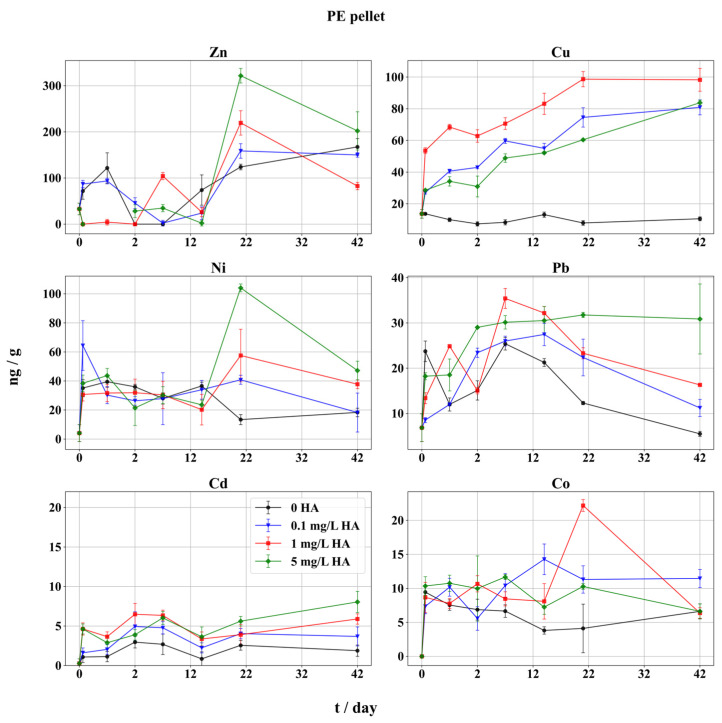
Adsorption of trace metals on PE pellets in seawater in presence of HA. Adsorbed metal fractions (expressed as ng metal/g fibers) with 95% confidence intervals. Symbols indicate different HA concentrations: black circles represent metal adsorption without HA (0 mg/L HA), blue triangles indicate 0.1 mg/L HA, red squares indicate 1 mg/L HA, and green diamonds represent 5 mg/L HA. The x-axis is scaled unevenly for readability, with a finer scale for the initial 2 days, followed by a broader scale for the period from 2 to 42 days.

All the adsorbed metals on PE pellets show a pattern of distinctive behavior during the various stages of the experiment (Figure 2), generally following the sorption order of Zn > Cu > Pb > Ni > Co~Cd when exposed to higher concentrations of HA (1 and 5 mg/L). This pattern corresponds to the general order of metal concentrations in seawater [49], and is consistent with previously reported adsorption behaviors of metals on plastics in marine and estuarine environments [20,58]. Moreover, the order of adsorption presented here was similar to that seen in the study involving biofilm adsorption, with the highest quantities of Cu, and lowest of Co [14].

Correlations are performed separately for all three types of samples (PE pellets, PP pellets, and fibers) and results are presented as correlation matrix in Table S2. It could be observed that in each of three types of samples a large number of statistically significant correlations exist. In the dataset consisting of PE pellets, Cd and Pb show statistically significant correlations with HA concentration, which would mean that it influences adsorption. The regression analysis confirmed that changes in HA concentration significantly influence the adsorption of Cd and Pb on PE pellets (Chapter S2).

Lead and Cu had similar adsorption dynamics throughout the experiment, while different behavior was noticed for Zn, Ni, Cd, and Co. In this study, no modeling experiments were performed to obtain the adsorption rates; however, this will be considered in future investigation. The calculated speciation of TM in seawater (Table 2) shows a pattern where Cu and Pb are significantly present in seawater as organic complexes (99.7% of Cu and 53.6% of Pb at 5 mg/L HA), indicating the adsorption onto MPs is strongly influenced by the speciation of metals, especially in the form of organic complexes, under natural seawater conditions.

The lowest adsorption of Pb and Cu was visible at the concentration 0 mg/L HA (Figure 2). This is in agreement with the study [13] that showed adsorption of Cu and Pb on virgin PE and PP without organic matter, where both polymers exhibited a weak preference for metal adsorption, presumably due to a lack of organic ligands in the solution. Also, the adsorption of 55 metals on PE and PET microplastics at neutral pH without organic ligands was reported [19]. Their findings were consistent with this study, revealing that Cu and Pb adsorbed to MP only to a small extent.

Cadmium exhibited the weakest adsorption of all metals on PE pellets (Figure 2). Speciation analysis (Table 2) confirmed that the majority of Cd in seawater is present in chloride form (over 90%, regardless of HA amounts), and only ~3.5% is found in free ionic form. However, regression analysis confirmed the influence of HA concentration on Cd adsorption, probably due to a weak Cd-HA complex formation (3.4%, Table 2) with the ligand addition of 5 mg/L HA. In the seawater, Cd is poorly complexed by organic ligands [59], and its toxicity and availability decrease in media of higher salinity [60]. The increased ionic strength of seawater, compared to diluted model solutions used in earlier MP adsorption studies, will enhance competition of metals for adsorption sites and the chloride complexation will reduce activity of free Cd, strongly inhibiting its adsorption onto MPs and possibly prolonging the adsorption equilibration [61]. Other authors also found the weakest adsorption of Co and Cd on virgin and beached PE [7]. On the other hand, a particular enrichment of PE pellets with Cd was noticed [6], indicating possible complexation of Cd in seawater with organic ligands, which do not belong to the humic class [62,63].

The adsorption equilibrium of Cd was reached after 21 d (Figure 2). At the end of the experiment, adsorbed Cd increased twofold compared to the values measured after the first sampling (3 h). The most Cd adsorbed on MPs was in the sample with 5 mg/L of HA, and the least was seen in the blank sample. The same trend was observed with Pb and Ni on PE MPs (Figure 2). Only Cd and Pb, on PE, had a rapid increase in adsorbed mass during the first 2 d of the experiment. However, in the second stage of the experiment Pb exhibited sustained decreases, except with the highest level of HA, where the mass of Pb remains constant. Similar adsorption kinetics in seawater were measured on aged and virgin MPs, while within 2 d large increases in Cu and Cd were found, particularly on aged MPs [18]. The same authors found the adsorption rate was highest in the first 5 h of the experiment, followed by a slow increase for the next 20 h. However, they did not report a decrease in the mass of adsorbed metals, in contrast to this study (in the presence of HA). It could suggest a role of HA in metal desorption by stabilizing complexed metals in the seawater. Some previous studies also observed that metals could start to desorb after adsorption peaked [64].

**Figure 3:**
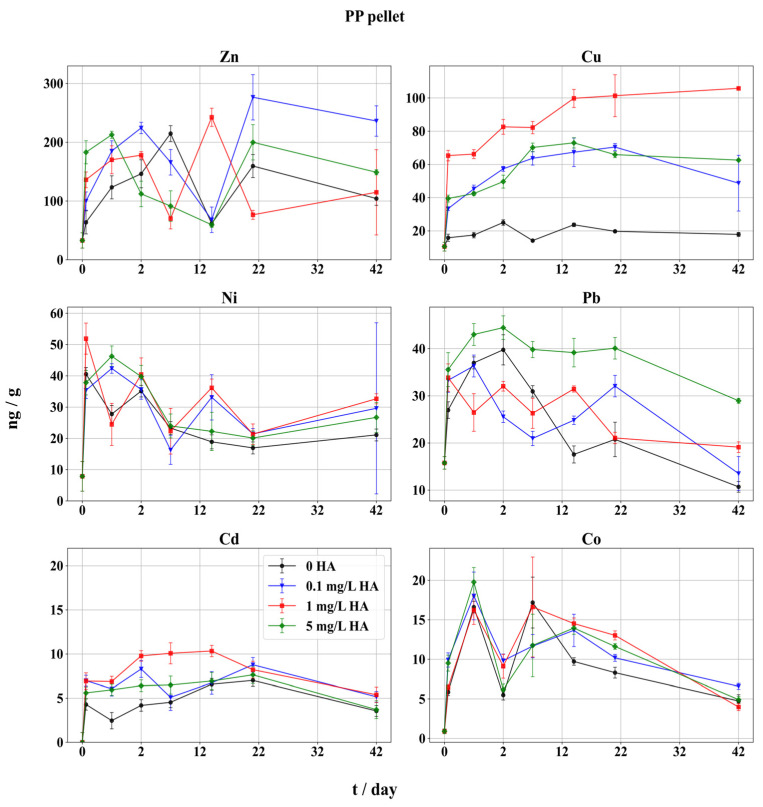
Adsorption of trace metals on PP pellets in seawater in presence of HA. Adsorbed metal fractions (expressed as ng metal/g fibers) with 95% confidence intervals. Symbols indicate different HA concentrations: black circles represent metal adsorption without HA (0 mg/L HA), blue triangles indicate 0.1 mg/L HA, red squares indicate 1 mg/L HA, and green diamonds represent 5 mg/L HA. The x-axis is scaled unevenly for readability, with a finer scale for the initial 2 days, followed by a broader scale for the period from 2 to 42 days.

The calculated speciation of Pb (Table 2) coincides with HA at 5 mg/L (53% of Pb in the HA-Pb complex), showing that Pb prefers to bind to PE particles in the organic form. Earlier, authors found critical concentrations needed for the complexation of several metals with marine humic substances in seawater [51]. It was determined that the complexation of Cd and Pb reaches only 10% at 0.4 mg/L of HA, and for Zn at ~1 mg/L. The same authors found that for levels of HA below 0.4 mg/L the complexation of Zn in seawater did not occur. Accordingly, in this study the samples contained 0.1 mg/L HA, which was probably too low for the significant organic complexation of Cd and Zn. The speciation analysis (Table 2) also indicates a major increase in the HA-Cu complex in the solution with 1 mg/L of HA or higher. This Cu behavior in solution supports the one observed for Cu adsorption, with the noticeable increase in Cu’s adsorbed amounts in the initial stage of the experiment. The amount of adsorbed Cu increased almost linearly until the equilibrium was reached after 21 d (Figure 2). Earlier, it was found that adsorbed amounts of metals from seawater on MPs would reach equilibrium (in the absence of HA) already after 6 and 9 d for Cu and Zn, respectively, indicating that metal-HA formation might decrease the rate of metal adsorption, due to organic complex stabilization in seawater [11]. According to the maximum adsorbed fractions of metals (Figure 2), the highest values for Zn, followed by Cu, on both types of pellets were recorded (Figure 2 and Figure 3). This is due to the high stability of the HA-Cu complex, which keeps a significant fraction of total Cu in dissolved organic form [65,66]. However, Cu also exhibited the most evident impact of HA on its adsorption, as the mass of Cu in the blank sample (without HA) remained mostly constant during the experiment, while the samples containing HA showed a steady increase in adsorbed Cu. The difference in adsorbed Cu fractions at the end of the experiment is notable, with the least Cu (11 ng/g) adsorbed from the blank sample (without HA), but with almost an order of magnitude increase in adsorbed metal (81–98 ng/g) from all samples containing HA. According to the speciation modeling (Table 2), without HA, the major fraction of Cu (>80%) in seawater is bound in inorganic form, significantly reducing the availability of free ionic Cu^2^ and inhibiting its adsorption onto MP. In all samples with added HA, adsorbed Cu fractions at the end of the experiment were significantly enhanced compared to the start of the experiment. Lead followed this trend only at the highest HA concentration (5 mg/L), while the other samples, including the blank sample, showed a decrease in adsorbed metal, indicating a desorption of both organic complexed and inorganic Pb.

Copper exhibited strongest adsorption in the samples with 1 mg/L and 0.1 mg/L of HA, while the adsorption pattern of Pb followed the amounts of added HA. The copper adsorption behavior implies the formation of a stable HA-Cu complex in the seawater, which stays unbound to MPs. According to the HA pKa value, regularly below five for carboxylic groups and between seven and nine for phenolate [67], most carboxylic groups and some 50% of phenolate groups are deprotonated, making HA readily available for metal accumulation in the slightly basic medium used in this experiment (seawater, pH ~8), as the form of HA at a pH between 7.6 and 8.0 should be favorable for metal complexation. Furthermore, the pH increase makes the polymer surface (PE and PP) negatively charged, which increases the capacity for adsorption of metal cations [68], but at the same time might decrease the interaction of negatively charged HA with the polymer surface. Similarly, a strong decrease in Cu adsorption on MPs was reported when the highest concentration of FA was present in the solution, as the stable complexes preferred to stay in the dissolved form [31].

No decrease in Cu adsorption was observed at all HA concentrations. The steady increase in adsorbed Cu indicates the formation of a stable HA-Cu complex in the seawater, and a slow rate adsorption on the polymer surface. In accordance with Humic ion-binding model IV [69], Cu shows the highest stability constants for binding with HA, suggesting that the majority of Cu is adsorbed as a stable complex formed in the solution and not in a free, ionic form. The positive change in adsorbed Cu in the last phase of the experiment was notable only at the highest level of HA, and, contrary to all the other metals, a consistent increase was detected. This suggests that the Cu adsorption equilibrium was not reached after 42 d of experimentation, hence, Cu can further accumulate on MP particles if an abundant source of HA is available in the environment.

Zinc, Ni, and Co exhibited a similar adsorption pattern on PE pellets (Figure 2). In the first 14 d, varying adsorption behavior was observed, particularly for Zn and Ni. The HA addition produced a slight increase in mass of adsorbed Co, which was much less pronounced for the other two metals. The lowest level of HA had little influence on the adsorption of Zn and Ni, which is in accordance with the low available fraction of the metal–HA complex (Table 2). At 1 and 5 mg/L HA, a slight increase in adsorbed Zn and Ni was observed at the end of the experiment. A steep decrease in Zn, Ni, and Co adsorption occurred in the second half of the experiment, possibly due to desorption of their labile HA complexes from polymer surfaces. Samples with the lowest added HA (0.1 mg/L) did not exert such behavior, as after 21 h equilibrium was reached and the adsorbed metal fractions remained constant or slightly increased, similar to the blank samples. Additionally, Zn showed a unique adsorption pattern in the blank sample and the sample with 0.1 mg/L of HA, where the adsorption increased, almost reaching the amounts adsorbed in the sample with the highest HA, 5 mg/L, which was not recorded for other metals. The most probable mechanism could be that HA binds onto MPs prior to metal adsorption, which is indicated by slower and constant increases in Zn’s adsorbed amounts from the blank sample and the sample with 0.1 mg/L of HA (Figure 2). Due to hydrolysis processes under these experimental conditions, a certain fraction of the metal in seawater was not available for adsorption, and gradually precipitated, with approximately half of the Zn^2+^ converting to poorly soluble Zn(OH)_2_ at pH ~8 [23]. At this pH value, MPs showed preference for slow-rate adsorption of inorganic and precipitated Zn when HA was scarce. Additionally, according to species distribution (Table 2), only the highest level of HA causes a sharp rise in the Zn-HA fraction with a decrease in free ionic Zn^2+^.

The overall quantities of adsorbed Ni and Co were low, in contrast to some earlier studies [8] where significant adsorption of these metals on virgin and beached PE in estuarine ecosystems was measured. However, in the same report adsorption was largely favored in conditions of decreased salinity. Batch adsorption experiments in distilled water and seawater showed a very strong adsorption of Pb and Zn on PE MPs, and all the metals showed stronger adsorption in seawater when compared to distilled water, probably owing to the present DOM, except for Co, which might be caused by interference from other cations or stabilization by major anions [12]. According to the speciation modeling (Table 2), the speciation of Ni and Co was dominated by the free, ionic form (>70%), while a significant fraction was also bound to inorganic anions, suggesting a majority of Ni and Co amounts were adsorbed as inorganic species.

### 3.2. Adsorption on PP Pellets

The adsorption trend of metals on PP pellets was similar to that on PE pellets (Figure 3). In comparison to PE, the adsorbed Cd and Co fraction at the end of the PP experiment (Figure 3) were lower, while staying similar for Pb and Cu, and were slightly higher in the case of Ni and Zn. The order of adsorption on PP particles followed the PE pellets’ results: Zn > Cu > Pb > Ni > Co~Cd. This underlines the strong influence of speciation on the adsorption of these metals under natural conditions. The adsorption pattern was somewhat different for Cd, Ni, and Co, while it did not vary significantly for Pb, Zn, and Cu, compared to PE adsorption results (Figure 2), and the influence of added HA was most pronounced for Cu. Slightly different trends were found with PP particles in artificial lake water enriched with FA, where Pb was the most adsorbed, followed by Cu and Cd [70]. In the same paper, Cu adsorption was highest on PS particles, followed by Pb and Cd; however, the adsorption equilibrium was achieved after 72 h, which was not observed in this work. In aquatic environments, Cu forms stronger complexes with HA in comparison to Zn, Pb, and Cd, with increased stability in solutions of higher pH [71,72]. In this study, the adsorption of Cd was low on both PE and PP particles, while earlier a strong adsorption capability of PP for Pb, Cu, and Cd was found, but in conditions of reduced salinity, where chloride ions do not stabilize Cd in solution to a great extent [21].

Cu adsorption kinetics on PP particles had some similarities compared to PE. A steep increase was noticed within the first 24 h, which slowed down until day 14, when equilibrium was achieved (Figure 3). Contrary to the adsorption on PE, no increase in Cu after 21 d in the system with 5 mg/L of HA was registered. As with PE, the smallest amount of Cu was adsorbed in the blank sample (18 ng/L) with concentrations remaining constant throughout the experiment, while a threefold to sixfold increase (49–106 ng/g) of adsorbed metal was found in samples containing HA. Increased Cu adsorption was also observed with 1 mg/L HA, as with PE. However, the influence of HA was less pronounced on PP pellets, as, in the samples with 0.1 and 5 mg/L HA, only ~50% of Cu was found, when compared to the sample with 1 mg/L HA. As discussed in Section 3.1, the adsorption of Cu on PP and PE pellets was not the most pronounced in the sample with the highest amount of HA, which might be attributed to the slow adsorption of the HA-Cu complex or the inhibition of complex formation when 5 mg/L HA was added. Some reports on the distribution of dissociation rates of the HA-Cu complex showed stronger dissociation with increase in metal–HA ratio [73,74,75].

At the end of the PP experiment, the amount of adsorbed Pb was comparable to the initial amount only at 5 mg/L HA. However, all the final Pb quantities were lower compared to initial adsorbed Pb amounts (Figure 2). The regression analysis (Chapter S2) revealed that variations in HA concentration have a significant impact on Pb adsorption on PE pellets, as was confirmed with correlation between Pb and HA concentration (Table S2).

At all HA levels, the maximum adsorption of Pb was reached within 1 to 2 d, after which a decline followed until the end of the experiment. Previously, authors found that the equilibrium of Pb adsorption on MP was reached after only 3 h, without added DOM [76]. They also reported an increased proportion of adsorbed Pb when smaller Pb concentrations were present in the solution. This might well imply that after 2 d, desorption and precipitation of Pb occurred.

The blank sample (without HA) showed some differences in the first stage of the experiment, as the highest adsorption of Pb was achieved after 2 d, while in the PE experiment it took 7 d. It could be due to a different (faster) interaction between the PP polymer surface and inorganic or free ionic Pb species, compared to PE pellets. The highest fluctuation was observed in the sample with lowest HA (0.1 mg/L), while the sample with the 5 mg/L HA showed a constant trend, as in the PE experiment. In contrast to the reduced adsorption of Pb observed here, in some studies a more pronounced adsorption of Pb on MPs was found [13,16,17]. Moreover, the measurements of Pb in oceanic water also showed that over 50% of the Pb was strongly complexed by organic ligands [77]. However, pH influences the adsorption of Pb to a great extent, and precipitation was regularly observed in media with pH > 7 [78], especially if carbonates or sulfates are present [79,80].

The adsorption pattern of Zn follows large oscillations within the first 21 d of the experiment, when it stabilizes (Figure 3). These mass variations were more pronounced compared to PE pellets. The initial amounts of adsorbed Zn were proportional with added HA levels. This pattern was irregular in the case of PE, and it indicates that the Zn-HA complex prefers PP over PE, due to the surface charge of the polymer. The final adsorbed amounts of Zn, which were also slightly higher on PP, were in fact lower compared to the initial amounts, and contrary to the PE experiment.

A different adsorption behavior of Cd was found between PP and PE pellets. On PP polymer, the influence of added HA was notable only during the first 14 d. Similarly to PE, the highest amounts of Cd were found after 24 h at 1 mg/L HA. At other HA levels, adsorbed Cd increased slightly and reached the maxima until day 21, after which adsorbed Cd decreased in all samples, showing a minor influence of HA speciation on Cd adsorption (Table 2, Figure 3). Contrary to the PE experiment, adsorbed Cd amounts at the end of experiment were lower compared to initial results. Moreover, the highest adsorbed Cd corresponded to the samples with 0.1 and 1 mg/L HA, which agrees with Cd speciation distribution (Table 2). Different authors reported contradictory results, an increase in adsorbed Cd under the influence of HA [47], and HA promoted desorption of Cd from different types of polymer MPs [64]. The cobalt adsorption process on PP pellets (Figure 3) exhibited similar behavior to Cd, except the adsorption maxima were reached earlier, within 7 d, followed by consistent decrease. The variations in adsorbed amounts in the first half of the experiment were larger, particularly for Co on PP compared to PE, while at the first sampling adsorbed Co amounts did not follow the HA levels. Moreover, added HA had little influence on Co adsorption at the end of the experiment, as the adsorbed quantities were regularly lower than the initial quantities. Pronounced adsorption was found at 1 mg/L of HA; however, the final adsorbed Co amount was the highest at 0.1 mg/L HA, similar to the PE experiment. The Co speciation under marine conditions is under negligible HA influence (Table 2); therefore, Co in an inorganic form remains unchanged at all levels of HA, indicating a weak adsorption of almost exclusively inorganic Co from seawater.

Contrary to the PE results, the impact of HA on the adsorption of Ni on PP pellets was less expressed (Figure 3). The weakest adsorption of Ni in the second stage of the experiment was found in both the blank sample and at 5 mg/L HA, like Cd. The most pronounced Ni adsorption was achieved within 24 h. However, without HA and with 5 mg/L HA, a steady decrease in Ni through the experiment was recorded, while at 0.1 and 1 mg/L HA the Ni adsorption was approaching equilibrium after 21 d. At all HA levels a slight increase in Ni followed until the end of the experiment, which might indicate that the adsorption of inorganic Ni, as well as Co, is preferred, as their organic speciation in the seawater is negligible (Table 2). At the end of the experiment adsorbed Co amounts were lower on PP pellets in comparison with PE. Contrary to Co, Ni adsorption increased between 21 and 42 d. Saturation of the MP surface took more time to be reached for Ni after a quicker desorption step, possibly due to weaker preference for adsorption, which might be continued in a longer timescale experiment. Finally, the adsorption equilibrium was not reached (Figure 3). The process of adsorption and desorption in natural conditions takes place continuously, particularly for Co, as stated in [9]. The same authors also reported that a slow increase in adsorption continued for a prolonged time, and Co did not reach the saturation even after 1 year on HDPE and LDPE, indicating high adsorption capacity of these polymers and a complex equilibrium under the influence of DOM.

### 3.3. Adsorption on PP Fibers

**Figure 4:**
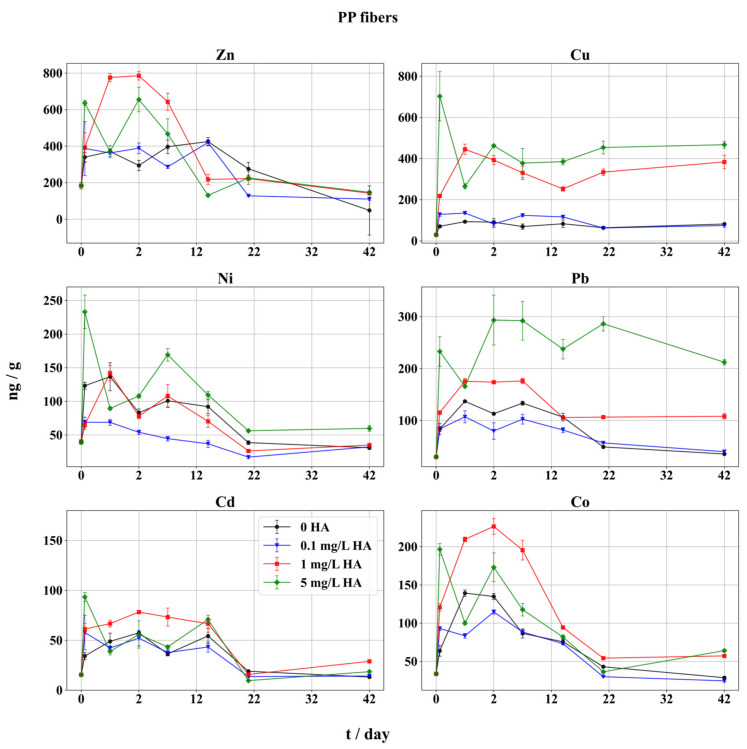
Adsorption of metals on PP fibers in seawater in presence of HA. Adsorbed metal fractions (expressed as ng metal/g fibers) with 95% confidence intervals. Symbols indicate different HA concentrations: black circles represent metal adsorption without HA (0 mg/L HA), blue triangles indicate 0.1 mg/L HA, red squares indicate 1 mg/L HA, and green diamonds represent 5 mg/L HA. The x-axis is scaled unevenly for readability, with a finer scale for the initial 2 days, followed by a broader scale for the period from 2 to 42 days.

Comparatively, PP fibers showed an increased amount of adsorbed metals, in comparison with both PE and PP pellets, mostly due to fibers’ higher specific surface area (Figure 4). Notably, the adsorption sequence on PP fibers was slightly different from pellets: Cu > Zn > Pb > Co~Ni > Cd. The highest accumulation was recorded for Cu and Pb, up to 470 and 212 ng/g, respectively. On the contrary, adsorbed Zn amounts at the end of the fibers experiment were lower compared to Cu and Pb and compared to Zn adsorbed on PE and PP pellets. However, maximum Zn adsorption in the first 7 d was higher than found on pellets, with a steep decrease afterwards. A decrease was detected also for Ni and Co, which also performed better on fibers, while Cd was adsorbed very weakly, but in amounts significantly higher than on pellets (up to order of magnitude). The order of adsorption of metals on PP fibers was slightly different in comparison with both PE and PP pellets: Cu > Zn > Pb > Co~Ni > Cd, yet still with pronounced influence of marine speciation.

The differences between adsorbed metal quantities on fibers and pellets at the end of the experiments were the largest for Pb and Cu, with up to 8 and 5 times more adsorbed metal on fibers compared to pellets, respectively. Nickel was adsorbed on pellets and fibers in almost the same amounts. Adsorption trends of Cu, Pb, Co, and Cd in the blank sample (without HA) and the sample with the lowest HA (0.1 mg/L) showed almost identical behavior throughout the experiment. However, the blank sample had quite similar adsorption or even outperformed the sample with 0.1 mg/L of HA, showing the particular influence of the fibers’ large surface when HA in the sample was too low for significant complex formation. Although the fraction of the metal–organic complex at such low levels of HA is insignificant (Table 2), this behavior was not observed in the experiments with pellets. Clearly, the significantly higher specific surface area of the fibers takes precedence for metal adsorption over HA levels when the ligand amounts are low.

The regression analysis showed that changes in HA concentration significantly affect the adsorption of Pb, Cu, and Ni on PP fibers. Additionally, correlation analysis confirmed strong associations between HA concentration and the adsorption of Pb, Cu, and Ni (Chapter S2).

At the highest level of HA, adsorption of Pb and Cu showed a rapid increase within 48 h, while at lower levels of HA adsorption increased quickly, within 24 h. The amount of adsorbed Pb was the highest in all three types of tested MPs at the 5 mg/L HA level, which is in accordance with a significant formation of HA-Pb complex in seawater with increasing HA levels (Table 2). Contrary to this study, the higher initial rate increase in Cu adsorption on pellets was observed [45], where in the presence of the ligand the smaller PS particles with larger available surface areas had a higher rate of adsorption within the first 48 h. Additionally, in this work, the absolute values of the adsorbed Cu and Pb were significantly higher in the samples containing more HA, showing a strong influence of the ligand on adsorption on fibers. Similar results were obtained with adsorption of Pb on PS MPs [46].

Although Cu forms stable complexes with HA, some reports found the complex was labile in the presence of competitive major cations in seawater [35,81]. This implies that a higher concentration of HA is needed to reach the stability of the HA-Cu complex.

A similar adsorption pattern was recorded among Zn, Ni, Cd, and Co (Figure 4). For Cd and Ni, maximum adsorption was achieved in the first stage of the experiment. At 5 mg/L of HA for Cd, Ni, and Co, the strongest adsorption was found after 3 h. For Cd, Zn, and Ni, the maximum adsorption in the blank sample (without HA) was reached only after 14 d. Moreover, Cd and Co in the sample at 1 mg/L HA had the strongest adsorption within the first 14 d, just as in the PP pellets experiment. Yet, a decrease in adsorbed Cd at all HA concentrations occurred after 14 d, and of Co after 48 h. The complexed form of Zn is not strongly bound to fibers and stays in the solution, which might explain the rapid decrease in adsorption at 5 mg/L of HA. The final quantities of adsorbed Zn were regularly lower than the initial ones at zero-hour sampling and were similar between the samples. Small variations in adsorbed Zn, Ni, Cd, and Co were found after 21 d. Contrary to these results, it was found that MP readily adsorbed Pb, followed by Cu, Zn, and Cd, with higher amounts found on larger MPs [17]. Moreover, the same authors found that adsorption equilibrium was reached slower with larger MPs, which fits these results for Cd adsorption. Furthermore, Cd had the fastest desorption rate of all metals, and Zn showed the slowest adsorption kinetics [17], as was confirmed in this study.

The amounts of Cd adsorbed on fibers after 42 d were six- to sevenfold higher when compared to the pellets, while the difference in lowest and highest adsorbed Cd was ~35%, the lowest of all TMs. In general, the presence of HA had very little impact on speciation and adsorption of Cd on MP, with higher amounts of Cd found on fibers only due to the high specific surface area. However, a strong influence of HA on the adsorption of Cd on MPs was reported, and for it, equilibrium was reached after 24 h [47]. The same study reported the weakest adsorption on PP being that of Cd, while the specific surface area was described as being very influential for adsorption.

The adsorption trends of both Ni and Co on PP pellets bear more resemblance to PP fibers than to PE pellets, especially in the case of Ni. The amounts of Ni and Co adsorbed on fibers were regularly higher than on the pellets, mainly due to a larger available specific surface area. Up to an eightfold increase in adsorbed Co was noticed on fibers when compared to pellets due to surface availability. At the end of the experiment, the presence of HA showed a larger influence on adsorption in the case of Ni (only at the highest HA level) and Co (middle and highest HA level), while the lowest HA concentration did not result in enhanced adsorption.

Previous studies have examined the toxic effects of microplastics and TM by exposing organisms to solutions containing high concentrations of both toxicants. For instance, polystyrene MP (PSMP) has been observed to increase Cd accumulation in the liver, gut, and gills of zebrafish (*Danio rerio*) [82]. Combined exposure to PSMP and Cd have led to severe oxidative damage and inflammation in zebrafish and discus fish (*Symphysodon aequifasciatus*), with Cd concentrations of 10 g/L and 50 g/L, respectively [82,83]. Similar results were found in freshwater grass carp, with a combined effect of PSMP and Cd on accumulation of Cd and severe abnormalities in the intestines [84]. Although the amount of Cd adsorbed on microplastics in these studies was relatively low, the findings suggest that MPs can act as vectors for Cd entry into marine organisms, potentially causing harmful effects. Additionally, trace metals (Cd, Cu, Pb) adsorbed onto high-density PE (HDPE) microplastics have been linked to oxidative stress in the yellow seahorse (*Hippocampus kuda*), highlighting the significant impact of these pollutants on marine life [85].

While this study did not focus on toxicological experiments, it contributes to a better understanding of how organic matter may influence the adsorption of trace metals onto microplastics. The findings provide valuable context for future research into how microplastics could enhance the bioavailability and toxicity of trace metals in the marine environment. This research also complements existing toxicological studies by offering insights into the physical and chemical interactions between organic matter, trace metals, and microplastics in natural waters. Further studies should explore the specific role of organic matter in modulating trace metal adsorption onto microplastics and assess how these processes impact marine organisms over longer timescales. Future toxicological research could build on this study’s findings by investigating how variations in organic matter content influence the combined effects of microplastics and trace metals on different marine species, particularly in natural environmental conditions.

## 4. Conclusions

This study shows the importance of the formation of stable metal complexes on the interaction of trace metals and plastics under marine conditions. It is demonstrated that both organic and inorganic TM species significantly influence TM adsorption onto plastic materials in seawater. Specifically, our results reveal a pronounced impact of environmentally relevant concentrations of humic acid on the speciation of trace metals and their subsequent adsorption onto plastics. Speciation modeling supports these findings, showing that dissolved Cu and Pb predominantly associate with humic acids, enhancing their adsorption, while Cd, Co, and Ni mostly exist as free ions or inorganic complexes in solution, affecting their adsorption dynamics differently.

This work advances knowledge of the influence of organic matter on the dynamics of trace metal adsorption on plastics, particularly PP, PE, and fibers, in aquatic environments. By altering metal bioavailability, humic substances can significantly impact the biome and potentially increase toxicity. This occurs through the formation of MP-HS-TM complexes, which, due to the high specific surface area of MPs, can more easily enter the food chain, posing risks to organisms. These findings enhance the understanding of the synergistic effects of plastic and trace metal pollution on a global scale in marine ecosystems. Furthermore, the methodologies and results presented here are expected to inform future studies aimed at addressing the global challenge of environmental pollution and its associated consequences.

## Figures and Tables

**Table 1:** Seawater parameters of the control (blank) samples, containing various mass concentrations of HA (*γ* = 0.1 mg/L (I), 1 mg/L (II), or 5 mg/L (III)), before and at the end of the model experiments.

Sample	*γ* (HA), mg/L	pH	Salinity, ‰
Seawater (Ploče), before pellets experiment	-	8.0	39
Seawater (Ploče), at the end of pellets experiment			
Blank	0	7.6–7.9	39
I	0.1		
II	1		
III	5		
Seawater (Medveja Bay), before fiber experiment	-	8.1	36
Seawater (Medveja Bay), at the end of fiber experiment			
Blank	0	7.7–7.9	36
I	0.1		
II	1		
III	5		

**Table 2:** Calculated distribution (speciation) of significant dissolved trace metal (TM) species in the seawater after metal addition (10 µg/L each) with different humic acid (HA) concentrations. Ionic strength was calculated by the Visual MINTEQ 3.1 software, *I* = 0.7 mol/L. Input parameters: pH 7.80; t = 21 °C; major components (mol/L): Cl^−^ 0.55, carbonate species 0.002, SO_4_^2−^ 0.029, Na^+^ 0.48, K^+^ 0.01, Mg^2+^ 0.055, Ca^2+^ 0.011, Sr^2+^ 0.0008.

conc. HA_TOT_/mg/L	0	0.1	1	5	
TM	% TM Species of Dissolved Metal Concentration				TM Species
Co	75.30	75.28	75.14	74.44	Co^+2^
	5.04	5.04	5.03	4.99	CoCl^+^
	15.42	15.42	15.39	15.25	CoSO_4_ (aq)
	2.62	2.62	2.61	2.59	CoHCO_3_^+^
	-	0.02	0.22	1.13	Co-HA
Cd	3.44	3.43	3.41	3.32	Cd^+2^
	49.18	49.15	48.87	47.51	CdCl^+^
	46.28	46.25	45.99	44.71	CdCl_2_ (aq)
	-	0.06	0.64	3.41	Cd-HA
Cu	13.49	11.86	2.93	0.04	Cu^+2^
	9.19	8.08	1.99	0.03	CuOH^+^
	3.90	3.43	0.85	0.01	CuCl^+^
	3.13	2.75	0.68	0.01	CuSO_4_ (aq)
	67.11	59.03	14.56	0.22	CuCO_3_ (aq)
	-	12.05	78.30	99.68	Cu-HA
Ni	74.04	73.99	73.51	71.28	Ni^+2^
	4.12	4.12	4.09	3.97	NiCl^+^
	15.20	15.19	15.09	14.63	NiSO_4_ (aq)
	2.32	2.32	2.31	2.24	NiCO_3_ (aq)
	4.08	4.08	4.05	3.93	NiHCO_3_^+^
	-	0.07	0.72	3.72	Ni-HA
Pb	6.76	6.65	5.75	3.14	Pb^+2^
	3.91	3.85	3.32	1.81	PbOH^+^
	35.45	34.90	30.15	16.46	PbCl^+^
	17.44	17.17	14.84	8.10	PbCl_2_ (aq)
	7.77	7.65	6.61	3.61	PbCl_3_^−^
	2.47	2.43	2.10	1.15	PbCl_4_^−2^
	3.51	3.46	2.99	1.63	PbSO_4_ (aq)
	19.34	19.04	16.45	8.98	PbCO_3_ (aq)
	2.41	2.37	2.05	1.12	PbHCO_3_^+^
	-	1.56	14.95	53.58	Pb-HA
Zn	51.98	51.55	47.58	30.78	Zn^+2^
	22.06	21.88	20.19	13.06	ZnCl^+^
	4.18	4.15	3.83	2.48	ZnCl_2_ (aq)
	11.67	11.58	10.68	6.91	ZnSO_4_ (aq)
	2.53	2.51	2.31	1.50	ZnCO_3_ (aq)
	-	0.81	8.47	40.77	Zn-HA

## Data Availability

All data will be available upon request.

## Supplementary Materials

The following supporting information can be downloaded at https://www.mdpi.com/article/10.3390/toxics12110820/s1, Table S1: Basic statistical parameters (N, Mean, Median, Sum, Minimum, Maximum, Range, and Std. Deviation) for: (A) PE pellet dataset, (B) PP pellet dataset, (C) fibers dataset. Table S2: Correlation matrix for all three types of samples: (A) PE pellets: correlations (Statistika_PE-pelet.sta); marked correlations are significant at *p* < 0.05000, N = 32 (Casewise deletion of missing data); (B) PP pellets: correlations (Statistika_PP-pelet.sta); marked correlations are significant at *p* < 0.05000, N = 32 (Casewise deletion of missing data); (C) Fibers: correlations (Statistika_Fibers.sta); marked correlations are significant at *p* < 0.05000 N = 32 (Casewise deletion of missing data). Figure S1: Scatterbox with boxplot for PE pellets samples; Figure S2: Scatterbox with boxplot for PP pellets samples; Figure S3: Scatterbox with boxplot for PP fibers. Chapter S1. Boxplot method. Chapter S2. Multiple regression analysis with HA concentration as independent variable.
