# Supplementary material for: Influence of Organic Matter and Speciation on the Dynamics of Trace Metal Adsorption on Microplastics in Marine Conditions
Source: Toxics. 2024 Nov 16;12(11):820. doi: 10.3390/toxics12110820 (PMC11598581; doi:10.3390/toxics12110820)
Supplement: Supplementary file 1 [file toxics-12-00820-s001.zip › toxics-3215639-supplementary.pdf]

Supplementary Material for Rapljenović et al. 2024: Influence of the organic matter and speciation on the dynamics of trace metal adsorption on microplastics in marine conditions

Table S1. Basic statistical parameters (N, Mean, Median, Sum, Minimum, Maximum, Range and Std. Deviation) for: A) PE pellet dataset, B) PP pellet dataset, C) Fibers dataset.

A)

	Valid N	Mean	Median	Sum	Minimum	Maximum	Range	Std.Dev.
Zn (ng/g)	32	71.16531	33.84500	2277.290	0.000000	321.7400	321.7400	78.44128
Cd (ng/g)	32	3.33344	3.50500	106.670	0.270000	8.0500	7.7800	2.06947
Pb (ng/g)	32	19.48406	19.89500	623.490	5.500000	35.4300	29.9300	9.02819
Cu (ng/g)	32	43.17125	41.81500	1381.480	7.350000	98.6600	91.3100	29.14457
Ni (ng/g)	32	31.70375	30.56500	1014.520	4.110000	104.1100	100.0000	19.47347
Co (ng/g)	32	7.95875	7.93000	254.680	0.000000	22.1800	22.1800	4.48879

B)

	Valid N	Mean	Median	Sum	Minimum	Maximum	Range	Std.Dev.
Zn (ng/g)	32	133.0931	129.7850	4258.980	33.01000	276.6000	243.5900	69.81960
Cd (ng/g)	32	5.6288	6.2350	180.120	0.00000	10.3400	10.3400	2.83973
Pb (ng/g)	32	27.9684	27.9750	894.990	10.70000	44.4700	33.7700	9.56662
Cu (ng/g)	32	49.0275	49.1950	1568.880	10.52000	105.8000	95.2800	29.28218
Ni (ng/g)	32	27.3638	25.6050	875.640	7.89000	51.9200	44.0300	11.64346
Co (ng/g)	32	9.6650	9.7800	309.280	0.92000	19.7800	18.8600	5.39426

C)

	Valid N	Mean	Median	Sum	Minimum	Maximum	Range	Std.Dev.
Zn (ng/g)	32	337.1109	317.2050	10787.55	48.52000	785.7100	737.1900	193.5459
Cd (ng/g)	32	40.8947	40.5500	1308.63	9.63000	93.3900	83.7600	23.1508
Pb (ng/g)	32	125.4581	106.9550	4014.66	30.00000	293.5700	263.5700	79.3621
Cu (ng/g)	32	214.7725	126.3700	6872.72	29.89000	703.0400	673.1500	177.7490
Ni (ng/g)	32	76.1800	66.9750	2437.76	17.07000	233.1000	216.0300	47.5551
Co (ng/g)	32	91.9625	82.6650	2942.80	24.59000	226.5000	201.9100	57.5910

Table S2. Correlation matrix for all three types of samples: A) PE pellets. Correlations (Statistika_PE-pelet.sta) Marked correlations are significant at $p < .05000$ N=32 (Casewise deletion of missing data); B) PP pellets. Correlations (Statistika_PP-pelet.sta) Marked correlations are significant at $p < .05000$ N=32 (Casewise deletion of missing data); C) Fibers. Correlations (Statistika_Fibers.sta) Marked correlations are significant at $p < .05000$ N=32 (Casewise deletion of missing data).

A)

	HA concentration (mg/L)	Time/day	Zn (ng/g)	Cd (ng/g)	Pb (ng/g)	Cu (ng/g)	Ni (ng/g)	Co (ng/g)
HA concentration (mg/L)	1.00	-0.00	0.03	0.37	0.36	0.13	0.23	0.09
Time/day	-0.00	1.00	0.62	0.36	0.10	0.49	0.17	0.19
Zn (ng/g)	0.03	0.62	1.00	0.21	0.10	0.31	0.57	0.27
Cd (ng/g)	0.37	0.36	0.21	1.00	0.61	0.71	0.42	0.46
Pb (ng/g)	0.36	0.10	0.10	0.61	1.00	0.50	0.40	0.47
Cu (ng/g)	0.13	0.49	0.31	0.71	0.50	1.00	0.34	0.58
Ni (ng/g)	0.23	0.17	0.57	0.42	0.40	0.34	1.00	0.54
Co (ng/g)	0.09	0.19	0.27	0.46	0.47	0.58	0.54	1.00

B)

	HA concentration (mg/L)	Time/day	Zn (ng/g)	Cd (ng/g)	Pb (ng/g)	Cu (ng/g)	Ni (ng/g)	Co (ng/g)
HA concentration (mg/L)	1.00	-0.00	-0.03	0.01	0.48	0.18	0.06	0.03
Time/day	-0.00	1.00	0.19	0.12	-0.35	0.33	-0.12	-0.15
Zn (ng/g)	-0.03	0.19	1.00	0.51	0.36	0.31	0.43	0.44
Cd (ng/g)	0.01	0.12	0.51	1.00	0.41	0.74	0.50	0.60
Pb (ng/g)	0.48	-0.35	0.36	0.41	1.00	0.24	0.60	0.52
Cu (ng/g)	0.18	0.33	0.31	0.74	0.24	1.00	0.33	0.36
Ni (ng/g)	0.06	-0.12	0.43	0.50	0.60	0.33	1.00	0.36
Co (ng/g)	0.03	-0.15	0.44	0.60	0.52	0.36	0.36	1.00

C)

	HA concentration (mg/L)	Time/day	Zn (ng/g)	Cd (ng/g)	Pb (ng/g)	Cu (ng/g)	Ni (ng/g)	Co (ng/g)
HA concentration (mg/L)	1.00	-0.00	0.10	0.11	0.72	0.67	0.40	0.16
Time/day	-0.00	1.00	-0.54	-0.45	-0.07	0.10	-0.42	-0.46
Zn (ng/g)	0.10	-0.54	1.00	0.74	0.49	0.46	0.64	0.91
Cd (ng/g)	0.11	-0.45	0.74	1.00	0.46	0.48	0.69	0.87
Pb (ng/g)	0.72	-0.07	0.49	0.46	1.00	0.83	0.64	0.56
Cu (ng/g)	0.67	0.10	0.46	0.48	0.83	1.00	0.56	0.57
Ni (ng/g)	0.40	-0.42	0.64	0.69	0.64	0.56	1.00	0.69
Co (ng/g)	0.16	-0.46	0.91	0.87	0.56	0.57	0.69	1.00

Chapter S1. Boxplot method

Scatterbox with boxplots were constructed for each of three datasets (PE pellets, PP pellets and Fibers) separately, to get insight into statistical anomalies and distribution.

In PE pellets dataset there are several anomalies (Figure S1). Zn distribution in PE pellet dataset is not very regular, despite that all samples except PE pellets with 5 mg/L HA present in the seawater solution, sampled after 21 days, are in the range which does not present anomalies. Mentioned sample is outlier (anomaly of lower degree). Cd, Pb and Cu does not show any anomaly in PE pellet dataset and show rather normal distribution, especially Pb, which distribution is like an example of statistical normal distribution. Ni shows two anomalous values. One of them (PE pellets with 5 mg/L HA present in the seawater solution, sampled after 21 days) is extreme (anomaly of higher degree), while the other anomalous value (PE pellets with 0.1 mg/L HA present in the seawater solution, sampled after 3 hours) is an outlier. The extreme value of Ni is in the same sample in which is outlier for Zn. Co in PE pellet dataset has several negative outliers, but they will not be discussed due to the fact that they are values equal to 0, i.e. values under the detection limit or which were not analyzed, so they can be ignored. Co also has one positive extreme value in the sample PE pellets with 1 mg/L HA present in the seawater solution, sampled after 21 days.

All samples from the PP pellet group do not show any anomalous values and all elements within this dataset have normal distribution, which is for majority of elements perfectly regular (Figure S2).

In Fibers dataset only Zn and Ni show outliers (anomalies of lower degree) (Figure S3). No other element shows any anomaly. Zn shows 2 outliers, and these samples are fibers with 1 mg/L HA present in the seawater solution, sampled after 24 hours and fibers with 1 mg/L HA present in the seawater solution, sampled after 2 days. Ni shows 1 outlier in sample fibers with 5 mg/L HA present in the seawater solution, sampled after 3 hours.

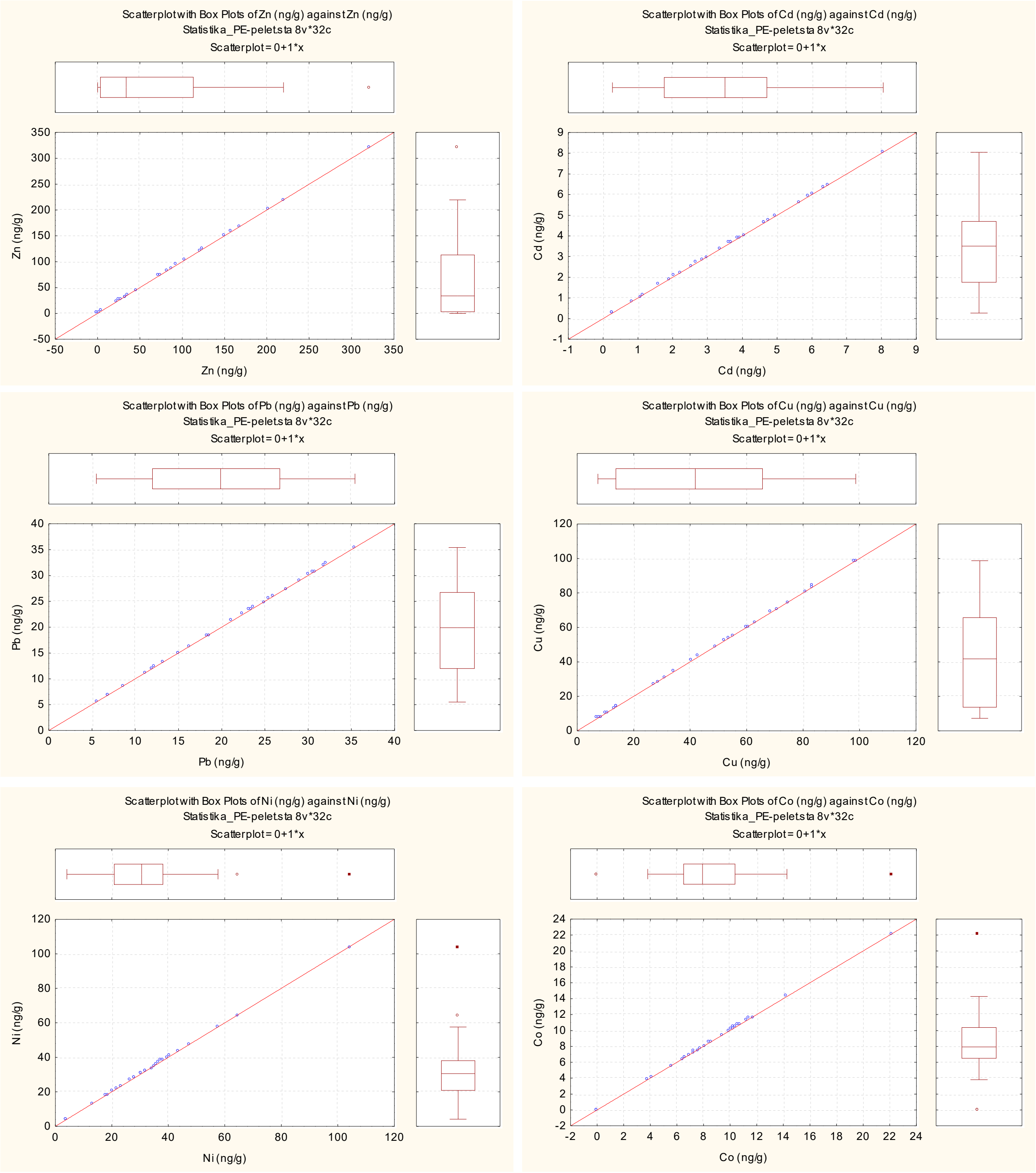

Figure S1. Scatterbox with boxplot for PE pellets samples.

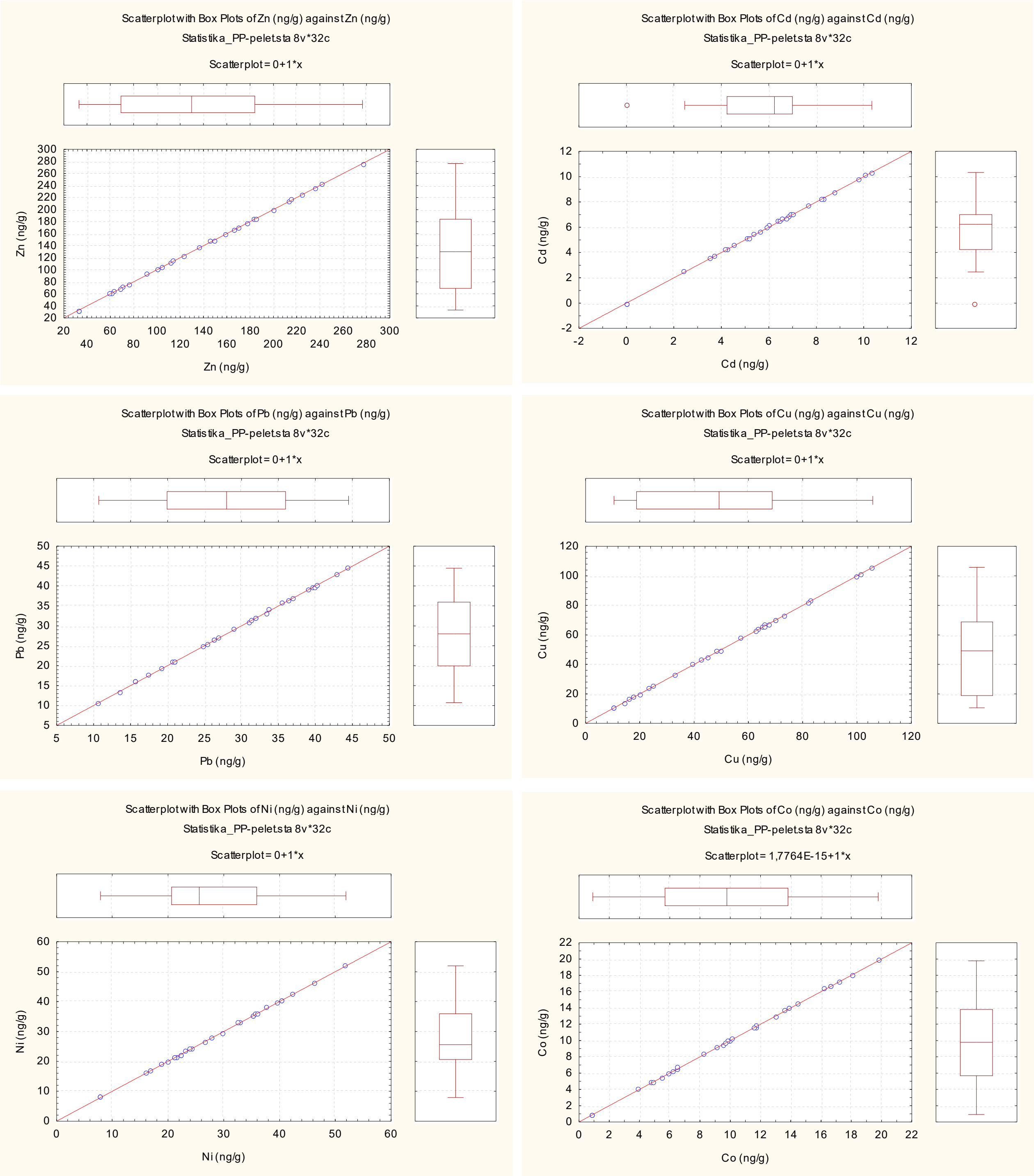

Figure S2. Scatterbox with boxplot for PP pellets samples.

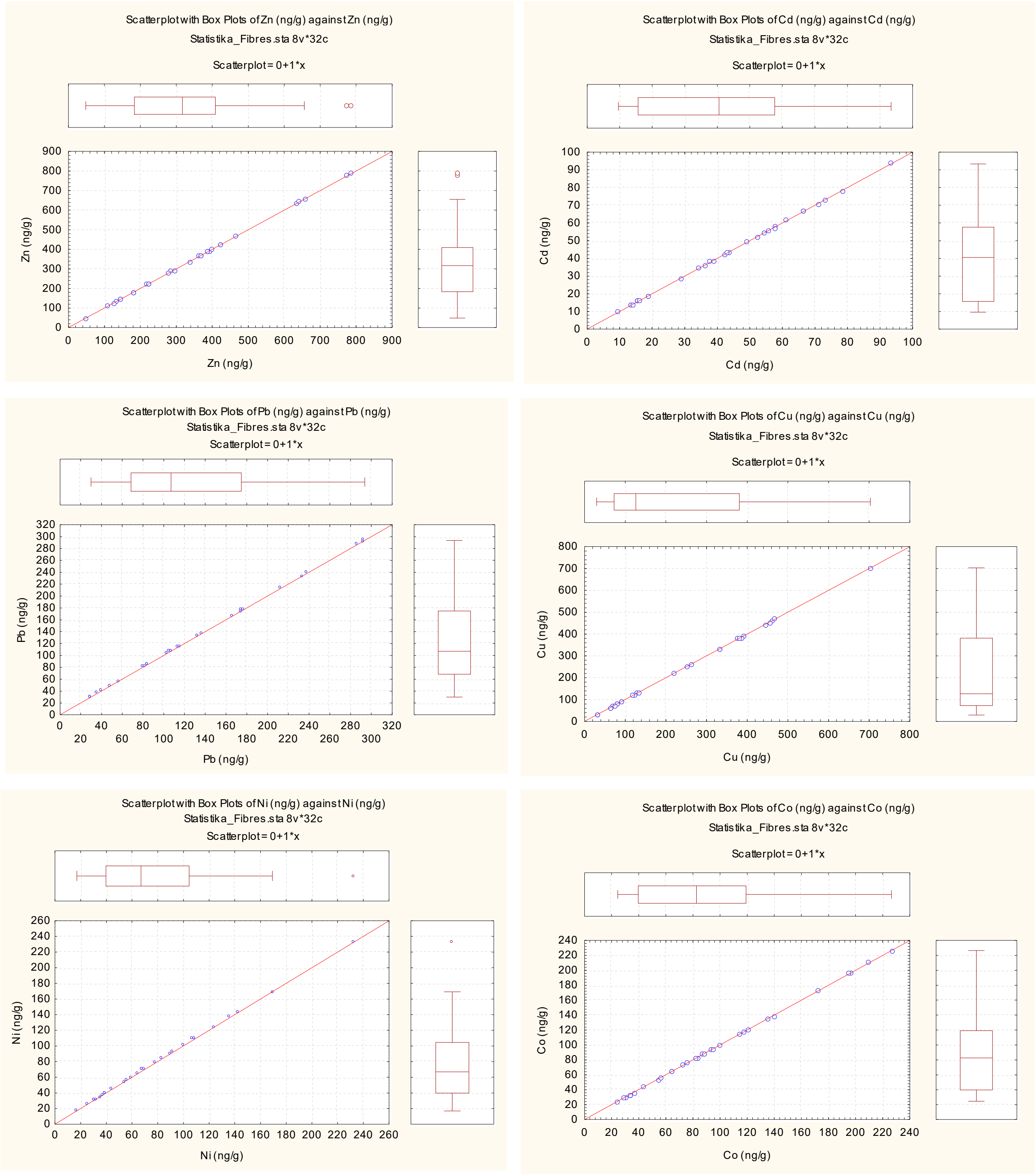

Figure S3. Scatterbox with boxplot for PP fibers.

Chapter S2. Multiple regression analysis with HA concentration as independent variable.

PE PELLET:

```
 Multiple Regression Results

Dependent: Zn (ng/g)          Multiple R =  .03298331     F = .0326725
                                      R2=  .00108790    df =    1.30
No. of cases: 32              adjusted R2= -.03220917     p =  .857775
             Standard error of estimate:79.694534346
Intercept: 69.265158050  Std.Error: 17.57794  t(   30) = 3.9405  p =  .0004
```

```
HA concentrat beta=.033


 Multiple Regression Results

Dependent: Cd (ng/g)         Multiple R =  .37099947     F = 4.788280
                                      R2=  .13764061    df =   1.30
No. of cases: 32             adjusted R2=  .10889529     p =  .036577
             Standard error of estimate: 1.953544153
Intercept:  2.769563445  Std.Error: .4308863  t(   30) = 6.4276  p =  .0000

HA concentrat beta=.371


 Multiple Regression Results

Dependent: Pb (ng/g)         Multiple R =  .36185089     F = 4.519900
                                      R2=  .13093607    df =   1.30
No. of cases: 32             adjusted R2=  .10196727     p =  .041846
             Standard error of estimate: 8.555526983
Intercept: 17.084786024  Std.Error: 1.887062  t(   30) = 9.0536  p =  .0000

HA concentrat beta=.362


 Multiple Regression Results

Dependent: Cu (ng/g)         Multiple R =  .13104980     F = .5242245
                                      R2=  .01717405    df =   1.30
No. of cases: 32             adjusted R2= -.01558682     p =  .474654
             Standard error of estimate:29.370822458
Intercept: 40.366178737  Std.Error: 6.478218  t(   30) = 6.2311  p =  .0000

HA concentrat beta=.131


 Multiple Regression Results

Dependent: Ni (ng/g)         Multiple R =  .22837148     F = 1.650696
                                      R2=  .05215353    df =   1.30
No. of cases: 32             adjusted R2=  .02055865     p =  .208696
             Standard error of estimate:19.272253600
Intercept: 28.437607549  Std.Error: 4.250813  t(   30) = 6.6899  p =  .0000

HA concentrat beta=.228


 Multiple Regression Results

Dependent: Co (ng/g)         Multiple R =  .08751572     F = .2315434
                                      R2=  .00765900    df =   1.30
No. of cases: 32             adjusted R2= -.02541903     p =  .633873
             Standard error of estimate: 4.545486142
Intercept:  7.670236795  Std.Error: 1.002582  t(   30) = 7.6505  p =  .0000

HA concentrat beta=.088
```

(significant betas are highlighted)

PP PELLET:

```
 Multiple Regression Results

Dependent: Zn (ng/g)        Multiple R =  .03104432     F = .0289404
                                     R2=  .00096375    df =   1.30
No. of cases: 32            adjusted R2= -.03233746     p =  .866059
             Standard error of estimate:70.939514339
Intercept: 134.68500206  Std.Error: 15.64688  t(   30) = 8.6078  p =  .0000

HA concentrat beta=-.03
```

```
 Multiple Regression Results

Dependent: Cd (ng/g)        Multiple R =  .00748388     F = .0016803
                                     R2=  .00005601    df =   1.30
No. of cases: 32            adjusted R2= -.03327546     p =  .967574
             Standard error of estimate: 2.886592851
Intercept:  5.613141740  Std.Error: .6366856  t(   30) = 8.8162  p =  .0000

HA concentrat beta=.007
```

```
  Multiple Regression Results

Dependent: Pb (ng/g)        Multiple R =  .48078913     F = 9.019730
                                     R2=  .23115818    df =   1.30
No. of cases: 32            adjusted R2=  .20553012     p =  .005346
             Standard error of estimate: 8.527019891
Intercept: 24.590410183  Std.Error: 1.880774  t(   30) = 13.075  p =  .0000

HA concentrat beta=.481
```

```
 Multiple Regression Results

Dependent: Cu (ng/g)        Multiple R =  .17525470     F = .9506239
                                     R2=  .03071421    df =   1.30
No. of cases: 32            adjusted R2= -.00159532     p =  .337357
             Standard error of estimate:29.305524343
Intercept: 45.258527420  Std.Error: 6.463816  t(   30) = 7.0018  p =  .0000

HA concentrat beta=.175
```

```
 Multiple Regression Results

Dependent: Ni (ng/g)        Multiple R =  .06454020     F = .1254858
                                     R2=  .00416544    df =   1.30
No. of cases: 32            adjusted R2= -.02902905     p =  .725637
             Standard error of estimate:11.811253619
Intercept: 26.811847411  Std.Error: 2.605166  t(   30) = 10.292  p =  .0000

HA concentrat beta=.065
```

```
 Multiple Regression Results

Dependent: Co (ng/g)        Multiple R =  .03070324     F = .0283073
                                     R2=  .00094269    df =   1.30
No. of cases: 32            adjusted R2= -.03235922     p =  .867517
             Standard error of estimate: 5.480846213
Intercept:  9.543362824  Std.Error: 1.208891  t(   30) = 7.8943  p =  .0000

HA concentrat beta=.031
```

(significant betas are highlighted)

PP FIBERS:

```
 Multiple Regression Results

Dependent: Zn (ng/g)        Multiple R =  .09883283     F = .2959284
                                     R2=  .00976793    df =   1.30
No. of cases: 32            adjusted R2= -.02323981     p =  .590467
             Standard error of estimate:195.78196965
Intercept: 323.06226863  Std.Error: 43.18293  t(   30) = 7.4812  p =  .0000

HA concentrat beta=.099
```

```
 Multiple Regression Results

Dependent: Cd (ng/g)        Multiple R =  .11347336     F = .3913249
                                     R2=  .01287620    df =   1.30
No. of cases: 32            adjusted R2= -.02002792     p =  .536334
             Standard error of estimate:23.381492086
Intercept: 38.965341725  Std.Error: 5.157173  t(   30) = 7.5556  p =  .0000

HA concentrat beta=.113
```

```
 Multiple Regression Results

Dependent: Pb (ng/g)        Multiple R =  .72106194     F = 32.49093
```

```
                                         R2=  .51993032    df =   1.30
No. of cases: 32            adjusted R2=  .50392800     p =  .000003
             Standard error of estimate:55.896635250
Intercept: 83.430404010  Std.Error: 12.32892  t(   30) = 6.7670  p =  .0000

HA concentrat beta=.721
```

```
 Multiple Regression Results

Dependent: Cu (ng/g)        Multiple R =  .67375554     F = 24.93967
                                     R2=  .45394653    df =   1.30
No. of cases: 32            adjusted R2=  .43574475     p =  .000024
             Standard error of estimate:133.51959108
Intercept: 126.81770855  Std.Error: 29.44994  t(   30) = 4.3062  p =  .0002

HA concentrat beta=.674
```

```
 Multiple Regression Results

Dependent: Ni (ng/g)        Multiple R =  .39506511     F = 5.548243
                                     R2=  .15607644    df =   1.30
No. of cases: 32            adjusted R2=  .12794565     p =  .025232
             Standard error of estimate:44.408782404
Intercept: 62.382013130  Std.Error: 9.795088  t(   30) = 6.3687  p =  .0000

HA concentrat beta=.395
```

```
 Multiple Regression Results

Dependent: Co (ng/g)        Multiple R =  .15549587     F = .7433422
                                     R2=  .02417897    df =   1.30
No. of cases: 32            adjusted R2= -.00834840     p =  .395432
             Standard error of estimate:57.830872004
Intercept: 85.385569542  Std.Error: 12.75555  t(   30) = 6.6940  p =  .0000

HA concentrat beta=.155
```

(significant betas are highlighted)
